# Brain volumetric changes in menopausal women and its association with cognitive function: a structured review

**DOI:** 10.3389/fnagi.2023.1158001

**Published:** 2023-09-25

**Authors:** Nur Zuliani Ramli, Mohamad Fairuz Yahaya, Nur Azlina Mohd Fahami, Hanani Abdul Manan, Meharvan Singh, Hanafi Ahmad Damanhuri

**Affiliations:** ^1^Department of Biochemistry, Faculty of Medicine, Universiti Kebangsaan Malaysia, Kuala Lumpur, Malaysia; ^2^Department of Biomedical Sciences, Faculty of Medicine & Health Sciences, Universiti Malaysia Sabah, Kota Kinabalu, Malaysia; ^3^Department of Anatomy, Faculty of Medicine, Universiti Kebangsaan Malaysia, Kuala Lumpur, Malaysia; ^4^Department of Pharmacology, Faculty of Medicine, Universiti Kebangsaan Malaysia, Kuala Lumpur, Malaysia; ^5^Functional Image Processing Laboratory, Department of Radiology, Faculty of Medicine, Universiti Kebangsaan Malaysia, Kuala Lumpur, Malaysia; ^6^Department of Cell and Molecular Physiology, Stritch School of Medicine, Loyola University Chicago, Maywood, IL, United States

**Keywords:** menopause, MRI, cognition, women, structural imaging, brain

## Abstract

The menopausal transition has been proposed to put women at risk for undesirable neurological symptoms, including cognitive decline. Previous studies suggest that alterations in the hormonal milieu modulate brain structures associated with cognitive function. This structured review provides an overview of the relevant studies that have utilized MRI to report volumetric differences in the brain following menopause, and its correlations with the evaluated cognitive functions. We performed an electronic literature search using Medline (Ovid) and Scopus to identify studies that assessed the influence of menopause on brain structure with MRI. Fourteen studies met the inclusion criteria. Brain volumetric differences have been reported most frequently in the frontal and temporal cortices as well as the hippocampus. These regions are important for higher cognitive tasks and memory. Additionally, the deficit in verbal and visuospatial memory in postmenopausal women has been associated with smaller regional brain volumes. Nevertheless, the limited number of eligible studies and cross-sectional study designs warrant further research to draw more robust conclusions.

## 1. Introduction

Menopause, a condition unique to women, typically occurs during the fifth decade of life and is diagnosed retrospectively 12 months after cessation of menstruation (Shuster et al., 2010). Clinically, it can be confirmed by evaluating the circulating levels of follicle-stimulating hormone (FSH) and/or anti-Müllerian hormone (AMH; Santoro and Johnson, 2019). The decline in the ovarian follicular reserve to a very low threshold is the main driver of the menopausal transition during midlife (Broekmans et al., 2004). During the perimenopausal stage, the menstrual cycle length becomes irregular, accompanied by changes in circulating estrogens and progesterone (Taffe and Dennerstein, 2002). It is common for the estrogen levels to fluctuate and be unpredictable but eventually reach a steady low state after completion of the menopausal transition (Hall, 2015). A scientific consensus in 2011 has led to the development of a gold standard criterion to distinguish the progress in the menopausal transition defined by the Stages of Reproductive Aging Workshop + 10 (STRAW + 10) based on the menstrual cycle pattern changes and FSH levels (Harlow et al., 2012).

While menopause is a normal biological process, the physiological changes during this critical period can be challenging to the individual. Diminished gonadal steroid hormone certainly has implications in the central nervous system, as many hallmark manifestations of menopause are neurological in nature, including forgetfulness, insomnia, depression, subjective memory complaints, and cognitive decline (Giannini et al., 2021). The brain is an important target for gonadal hormone effects. Localization of the classical estrogen receptors (ERα/β) and progesterone receptors (PRA/B) have been reported in regions important for cognition, including the hippocampus (Guerra-Araiza et al., 2003; Bean et al., 2014; Scudiero and Verderame, 2017), the medial prefrontal cortex (Brinton et al., 2008; Almey et al., 2014), the basal forebrain (Hammond and Gibbs, 2011), and the striatum (Enterría-Morales et al., 2016). Estrogen and progesterone have been regarded as neuroprotective hormones. Estrogen mediates morphological and neurochemical changes of the neural processes by stimulating brain-derived neurotrophic factor (BDNF; Luine and Frankfurt, 2013) and by transcription factors (Arevalo et al., 2015), cell signaling (Yang et al., 2020), neuronal growth (Scharfman and MacLusky, 2006; Bustamante-Barrientos et al., 2021), dendritic spine densities (Handley et al., 2022), synaptic organization (Wang et al., 2018), and regulation of cholinergic systems (Kwakowsky et al., 2016). Similarly, progesterone induce neuroprotection by activating the mitogen-activated protein kinase (MAPK) and protein kinase B (Akt) signaling pathways (Singh and Su, 2013; Liu et al., 2022), inhibiting excitotoxicity (Luoma et al., 2011), promoting myelin repair (Engman et al., 2018), and exerting anti-inflammatory effects (Aryanpour et al., 2017; De Nicola et al., 2018). Several studies have demonstrated the detrimental effects of menopause on cognitive function (Sullivan and Fugate, 2001; Epperson et al., 2013; Weber et al., 2014; Lee et al., 2020). Among the cognitive domains affected are attention, working memory (Maki et al., 2021), verbal memory (Kilpi et al., 2020), and executive function (Wegesin and Stern, 2007). Nevertheless, analysis from the Study of Women’s Health Across the Nation (SWAN) cohort reported cognitive decline only during the perimenopausal stage, and these changes were reversed during postmenopause (Greendale et al., 2009). Specifically, there is impairment in verbal memory scores during early and late perimenopause, while there is a lack of improvement in speed processing in the late perimenopausal phase compared with the pre- and postmenopausal phases (Greendale et al., 2009). However, there has been insufficient research to determine whether the detrimental effects of the menopausal transition on cognitive functions are time limited. To ascertain whether the cognitive decline is transient or permanent, it would be useful to replicate these findings in studies with a larger number of repeated assessments over a wider age span.

Neuroimaging studies using MRI in women have revealed that fluctuations in gonadal hormones at different reproductive stages could influence region-specific structural changes in the brain (Rehbein et al., 2021). At puberty, circulating estrogen levels are positively correlated with gray matter (GM) volumes in the middle frontal, inferior temporal, middle occipital, and parahippocampal gyri (Neufang et al., 2009; Peper et al., 2009), and negatively correlated with volume changes in the prefrontal, orbitofrontal, parietal, temporal, and anterior cingulate cortices (Peper et al., 2009; Koolschijn et al., 2014). There are also apparent structural alterations across different menstrual cycle phases, pregnancy, and the postpartum period. As such, variations in hormonal levels during the menstrual phase affect the hippocampal (Protopopescu et al., 2008; Lisofsky et al., 2015) and amygdalar (Ossewaarde et al., 2013) volumes, and there are pregnancy-related changes in regions subserving social cognition (Hoekzema et al., 2017). Considering that endogenous gonadal hormones have been associated with changes in brain structure, researchers have studied the efficacy of hormone replacement therapy (HRT) on brain volumes at menopause and its correlation with cognitive status (Kantarci et al., 2016, 2018). Several modifying factors, including the time of initiation, duration, and type of HRT and the critical window hypothesis, are important to contemplate when determining the outcome of HRT on brain volumes.

Given that gonadal hormone levels affect an array of brain cellular, morphological, and organizational changes, it is foreseeable that there are volume changes in the brains of postmenopausal women. However, most of the research related to structural and morphological brain changes in postmenopausal women has focused on age or hormone treatment. Few studies have examined structural brain changes related to the individual neurobiological mechanisms of menopause. Thus far, there have been no comprehensive structured review on this area of research; hence, we aimed to address this gap in the present review. Our primary objective was to summarize the current literature on structural brain changes as measured by MRI from cross-sectional and longitudinal studies associated with the menopause status. Our secondary objective was to determine whether the structural brain differences correlate with cognitive performance. We hypothesize that the postmenopausal period is associated with smaller volumes of brain regions associated with memory and executive function. These areas include structures in the frontal, temporal, and hippocampal regions, which are associated with poor cognitive performance.

## 2. Methods

### 2.1. Literature search

An electronic literature search was performed to identify studies done on differences in the structural brain of postmenopausal women. Two online databases—Medline via Ovid Medline and SCOPUS—were searched for papers published from 1945 to March 2022. The search strategy was limited to human studies and used a combination of the following sets of keywords: women or female*; elder or old or aging or age*; menopaus*; stuctur*; MRI*.

### 2.2. Selection of research articles

The outcomes generated from the two databases were retrieved to screen for eligibility. The studies were selected if they meet the following inclusion criteria:

- Full research articles or original articles.
- Volumetric brain structure assessment was done using an MRI scanner.
- There was a single MRI session (cross-sectional) or multiple sessions (longitudinal).
- The participants did not have any medical conditions that affect the brain structure, including stroke, brain tumors, neurodegenerative diseases, or other medical illnesses that may significantly alter central nervous system functions.
- The study population consisted of menopausal women (either peri- or postmenopausal), and the healthy controls comprised premenopausal women and men of the same age, to have a fair evaluation of the structural brain differences.
- Published in the English language.

The exclusion criteria were:

- Narrative reviews, editorial, letters, dissertations, book chapters, books, conference proceedings, and lectures.
- Case–control studies, interventional studies, or animal studies.
- Studies using other imaging modalities such as single-photon emission computed tomography, diffusion tensor imaging (DTI), and magnetoencephalography.
- The participants had neurological disorders, such as dementia, Alzheimer’s disease (AD), schizophrenia, and depression.
- Studies without a premenopausal group and men as a comparison.
- Use of HRT as an intervention in clinical trials.

### 2.3. Data extraction and management

In the first phase, three independent reviewers screened all the titles of the articles and removed articles that were unrelated to the study. In the second phase, the reviewers removed duplicate articles from the two databases and obtained the abstracts of the remaining articles. For the third phase, the reviewers assessed the abstracts based on the inclusion and exclusion criteria. Finally, the reviewers obtained the full text of the remaining articles and determined whether they could be included. All reviewers agreed to the inclusion of the final articles in the review. Any disagreements were resolved through consensus. The reviewers extracted data from the articles and entered it into evidence tables. The extracted data were the study title, the publication date, the research design (cross-sectional or longitudinal MRI), participant characteristics (e.g., age, biological sex, and percentage of menopausal women), MRI measures [e.g., GM, white matter (WM), and regional brain volumes], findings (e.g., differences observed between groups and adjustment for covariates), and neuropsychological measures.

### 2.4. Assessing the quality of the studies

Fourteen included articles were qualitatively assessed using the Quality Assessment Tool for Observational Cohort and Cross-Sectional Studies designed by the National Institutes of Health (NIH; National Institutes of Health, 2021). Quality assessment tools are needed to assess the internal validity of research findings, which is recognized as a risk of bias by the Cochrane Collaboration (Boutron et al., 2021). The tool consists of 14 questions corresponding to various aspects of study validity, including the research question, the population definition, the participation rate, recruitment, data collection, the sample size, analyses, outcome measures, and confounders. The questions are shown in Table 1. Two reviewers (HAD and NZR) judged the quality of the studies by rating each article as good (low risk of bias), fair (moderate risk of bias), or poor (high risk of bias). Finally, the assessments given by the two reviewers were compared to reach a consensus.

**Table 1:** Quality assessments of the included studies.

	Q1	Q2	Q3	Q4	Q5	Q6	Q7	Q8	Q9	Q10	Q11	Q12	Q13	Q14	Quality rating (good, fair, or poor)
den Heijer et al. (2003)	Yes	Yes	Yes	Yes	NR	NA	NA	NA	NR	NA	Yes	Yes	NA	Yes	Fair
Sullivan et al. (2005)	Yes	Yes	NR	Yes	NR	NA	NA	NA	NR	NA	Yes	NR	NA	Yes	Fair
Cowell et al. (2007)	Yes	Yes	Yes	Yes	NR	NA	NA	NA	NR	NA	Yes	CD	NA	Yes	Fair
Goto et al. (2011)	Yes	Yes	Yes	Yes	NR	NA	NA	NA	Yes	NA	Yes	NR	NA	Yes	Good
Mosconi et al. (2017)	Yes	Yes	NR	Yes	NR	NA	NA	Yes	Yes	NA	Yes	NR	NA	Yes	Fair
Lu et al. (2018)	Yes	Yes	NR	Yes	NR	NA	NA	NA	Yes	NA	Yes	Yes	NA	Yes	Fair
Kim et al. (2018)	Yes	Yes	NR	Yes	NR	NA	NA	NA	Yes	NA	Yes	NR	NA	Yes	Fair
Mosconi et al. (2018)	Yes	Yes	Yes	Yes	NR	Yes	Yes	Yes	Yes	Yes	Yes	NR	Yes	Yes	Good
Baek et al. (2019)	Yes	Yes	NR	Yes	NR	NA	NA	NA	Yes	NA	Yes	NR	NA	NR	Fair
Seitz et al. (2019)	Yes	Yes	Yes	Yes	NR	NA	NA	Yes	Yes	NA	Yes	NR	NA	Yes	Fair
Rahman et al. (2020)	Yes	Yes	Yes	Yes	NR	NA	NA	Yes	Yes	NA	Yes	NR	NA	Yes	Fair
Than et al. (2021)	Yes	Yes	NR	Yes	NR	NA	NA	NA	Yes	NA	Yes	yes	NA	Yes	Fair
Schelbaum et al. (2021)	Yes	Yes	Yes	Yes	NR	NA	NA	Yes	Yes	NA	Yes	NR	NA	Yes	Fair
Zhang et al. (2021)	Yes	Yes	NR	Yes	NR	NA	NA	NA	Yes	NA	Yes	NR	NA	Yes	Fair

Q1: Was the research question or objective in this paper clearly stated?; Q2: Was the study population clearly specified and defined?; Q3: Was the participation rate of eligible persons at least 50%?; Q4: Were all the subjects selected or recruited from the same or similar populations (including the same time period)? Were the inclusion and exclusion criteria for being in the study prespecified and applied uniformly to all participants?; Q5: Was a sample size justification, power description, or variance and effect estimates provided?; Q6: For the analyses in this paper, were the exposure(s) of interest measured prior to the outcome(s) being measured?; Q7: Was the timeframe sufficient so that one could reasonably expect to see an association between exposure and outcome if it existed?; Q8: For exposures that can vary in amount or level, did the study examine different levels of the exposure as related to the outcome (e.g., categories of exposure, or exposure measured as continuous variable)?; Q9: Were the exposure measures (independent variables) clearly defined, valid, reliable and implemented consistently across all study participants?; Q10: Was the exposure(s) assessed more than once over time?; Q11: Were the outcome measures (dependent variables) clearly defined, valid, reliable and implemented consistently across all study participants?; Q12: Were the outcome assessors blinded to the exposure status of participants?; Q13: Was loss to follow-up after baseline 20% or less?; Q14: Were key potential confounding variables measured and adjusted statistically for their impact on the relationship between exposure(s) and outcome(s)? CD: cannot determined; NA: not applicable; NR: not reported; Q: questions. Quality was rated as poor if the percentage of “yes” answers was ≤ 40%, fair (41–69%), and good (≥ 70%). Questions with “NA” as an answer were not counted in the total quality rating scores.

## 3. Results

### 3.1. Search results

**Figure 1:**
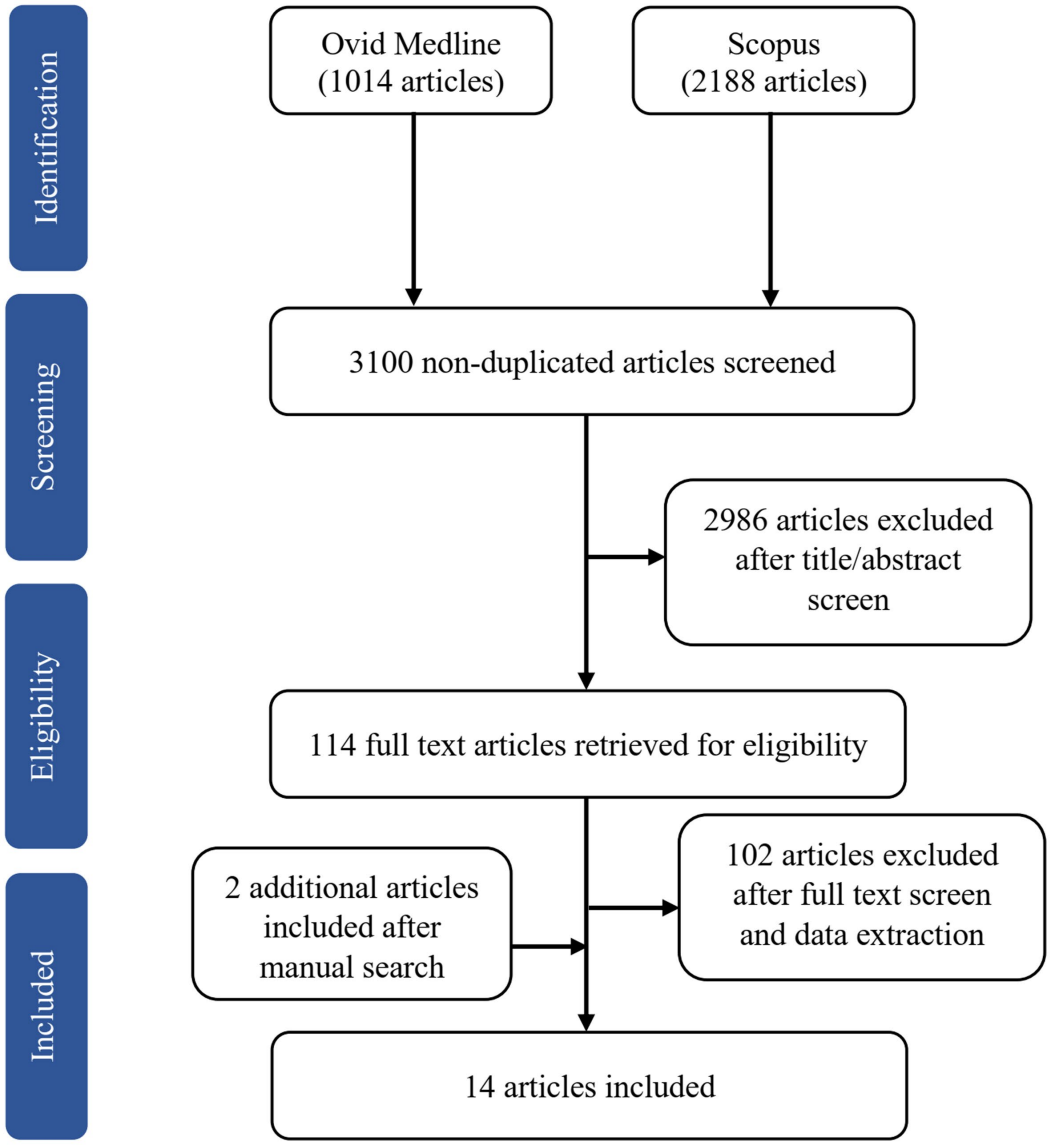
Flowchart illustrating the literature search and exclusion process.

The online search yielded 3,202 potentially relevant articles, of which 102 articles were duplicates. We excluded 2,986 articles after screening the titles and abstracts based on the inclusion and exclusion criteria. Of the 114 full-text articles retrieved, we removed 50 articles after reading the full text and another 52 after data extraction. The reasons for exclusion were studies used functional MRI (fMRI; *n* = 51), hormonal treatment as an intervention in clinical trials (*n* = 18), lack of comparison with a premenopausal group or men (*n* = 10), one article was from the same project as another study (*n* = 1), and irrelevant to the main objective (*n* = 22). We included two additional articles after a manual search of the SCOPUS database. Of the 14 articles included in this review, one is longitudinal (Mosconi et al., 2018), and the remaining are cross-sectional. Figure 1 shows the flow chart of study selection.

### 3.2. Quality assessments of the studies

The quality assessments of the included studies are shown in Table 1. We rated two studies as good (14%) and the rest as fair (86%). The cross-sectional study design in most of the studies does not allow identifying a causal association between the menopause status and brain volume. In addition, we noted a deficiency in the information regarding the statistical justification of sample size or estimates of the effect size. Furthermore, seven studies did not provide sufficient details on the participation rate; this increases the risk of selection bias, meaning that the study population may not be representative of the target population. Other limitations included underreporting whether the MRI rater was blinded to the participants’ menopause status. Most authors considered the effects of multiple potential confounders on the outcomes, including age, the type of menopause, the duration of menopause, HRT use, apolipoprotein E4 (ApoE4) carrier status, education level, and vascular risk factors. However, only three studies (Mosconi et al., 2017, 2018; Schelbaum et al., 2021) comprehensively controlled all these confounders, whereas the remaining studies marginally controlled several confounders. Indeed, the only eligible longitudinal study showed a high follow-up rate and adequate reassessment of the menopause status 3 years after baseline (Mosconi et al., 2018). In the current context, the level of exposure was regarded as the phase of menopause. When both peri- and postmenopausal women were present included in a study, there were apparent brain structure differences. The common strengths of most included studies were an accurate description of the study objective and population, uniformity in sample recruitment, and reliable staging of the menopause status.

### 3.3. Study descriptions

#### 3.3.1. Study population

The study population consisted of women at different reproductive stages, and five studies also included men (den Heijer et al., 2003; Mosconi et al., 2017, 2018; Rahman et al., 2020; Schelbaum et al., 2021; Table 2). The authors determined the postmenopausal population according to the STRAW+10 criteria (Mosconi et al., 2017, 2018; Kim et al., 2018; Lu et al., 2018; Baek et al., 2019; Seitz et al., 2019; Rahman et al., 2020; Schelbaum et al., 2021; Zhang et al., 2021), interviews (Goto et al., 2011), a self-reported questionnaire (Than et al., 2021), or unspecified criteria (den Heijer et al., 2003; Sullivan et al., 2005; Cowell et al., 2007). Some of the researchers measured additional sex steroid hormones, specifically FSH, to support the menopause status (Kim et al., 2018; Lu et al., 2018; Baek et al., 2019; Zhang et al., 2021). According to the STRAW +10 criteria, there are three reproductive phases in women, namely reproductive, the menopausal transition, and postmenopause, with a total of 10 stages centered around the final menstruation phase (FMP) at Stage 0 (Table 3). Each study included postmenopausal participants except for Lu et al. (2018) and Zhang et al. (2021), in which the target population was perimenopausal women. Due to a lack of in-depth menopause assessment, Cowell et al. (2007) consolidated peri- and postmenopausal women in a single group, whereas Than et al. (2021) combined pre- and perimenopausal women.

**Table 2:** Study descriptions.

	den Heijer et al. (2003)	Sullivan et al. (2005)	Cowell et al. (2007)	Goto et al. (2011)	Mosconi et al. (2017)	Lu et al. (2018)	Kim et al. (2018)	Mosconi et al. (2018)	Baek et al. (2019)	Seitz et al. (2019)	Rahman et al. (2020)	Than et al. (2021)	Schelbaum et al. (2021)	Zhang et al. (2021)
Study design	CS	CS	CS	CS	CS	CS	CS	LG	CS	CS	CS	CS	CS	CS
Study population	Post-M and Men	Post-M and Pre-M	Peri-M/Post-M merged and Pre-M	Post-M and Pre-M	Post-M, Peri-M, Pre-M, and Men	Peri -M and Pre-M	Post-M and Pre-M	Post-M, Peri-M, Pre-M, and Men	Post-M and Pre-M	Post-M, Peri-M, and Pre-M	Post-M, Peri-M, Pre-M, and Men	Post-M and Pre-M/Peri-M merged	Post-M, Peri-M, Pre-M, and Men	Peri-M and Pre-M
Adjusted for age?	Matched for age	No	No	No, age as a grouping factor at the sample level	Yes	Sig. brain differences between Peri-M and Pre-M were correlated with age to exclude age effects	Yes	Yes	No	Matched for age	Yes.	TBV: age and M interaction in subgroup analyses (<58 years old)	Yes	Matched for age
												RBV: age and M interaction		
Menopause assessment	-	-	-	Interview	STRAW +10 criteria	STRAW + 10 criteria	STRAW + 10 criteria	STRAW + 10 criteria	STRAW + 10 criteria	STRAW + 10 criteria	STRAW + 10 criteria	Self-reported questionnaires	STRAW + 10 criteria	STRAW + 10 criteria
Age at menopause	-	-	-	45.7 ± 2.9, 50.6 ± 3.1, 50.7 ± 4.3, and 49.6 ± 3.6 years for each Post-M in their 40, 50, 60, and 70 s, resp.	-	MT phase	-	-	-	-	-	-	51 ± 3 years	MT phase
Years after menopause	-	-	-	-	-	MT phase	5.5 ± 2.5 years	-	-	-	-	-	-	MT phase
Types of menopause	-	-	-	-	-	SM excluded	SM excluded	-	-	-	17% of Post-M and 4% of Peri-M had HYS	W > 60 years old with BO were regarded as Post-M	6% HYS, 6% pHYS, and 1% O	-
HRT use	HRT users excluded	41% of Post-M (active or past user)	46% of Post-M (active or past user)	-	14% of Post-M (active or past user)	HRT users excluded	HRT users excluded	HRT users excluded	HRT users excluded	HRT users excluded	26% of Post-M and 11% of Peri-M (active or past user)	7% of Post-M and 1.7% of Pre-M/Peri-M (active or past user)	4% past users and 27% current user	HRT users excluded
Education levels	1° ed. Of Post-M and Men was 37 and 23%, resp.	14.7 ± 3.6 years	≥ 13 years	-	≥ 12 years, adjusted	≥ 15 years	-	≥ 12 years	Post-M:	Post-M:	≥ 12 years	-	W: 17 ± 2 years	≥ 12 years
									1° ed. (26%)	HS (25%)				
									2° ed. (68%)	C (28%)				
									3° ed. (5%)	UNI (6%)				
									Pre-M:	Peri-M:			Men: 18 ± 2 years	
									2° ed. (68%)	HS (24%)				
									3° ed. (5%)	C (34%)				
										UNI (7%)				
										Pre-M:				
										HS (18%)				
										C (30%)				
										UNI (12%)				
CMR	BMI, smoking status, and alcohol consumption	-	-	No difference in DI, SI, and BG level, and HTN	No difference in BMI, HTN, WTH, BP, FG, and CHOL in all groups	-	-	No difference in HTN and BMI in all groups	-	Each group was matched for BMI	No difference in WTH, TG, and IR, except ↓ CHOL/HDL in Post-M compared with Pre-M and Peri-M	Post-M had ↑ no. of CMR compared with Pre-M/Peri-M	Smoking status, HTN, and WTH were adjusted	-
					Men had ↑ IR and ↑ TG with ↓ HDL:LDL ratio compared with women			Men had ↑ IR and ↑ CHOL/HDL compared with women			Men had ↑ IR and CHOL/HDL compared with women			
Brain regions investigated based on *a priori* hypothesis?	Yes, HIP	Yes, HIP and TL	Yes, PFC	Yes, HIP	Yes, FL (med., inf., and PFC), PCC, TL (lat. And med.), and PL (inf. And sup.)	No	No	Yes, HIP	No	Yes, HIP, DLPFC, ACC, IPL, and PHC	No	No	Yes, FL, TL, HIP, PHC, amygdala, fusiform gyrus, PCC, BA, and EC	Yes, SCV
ApoE4 carrier	27% in Post-M and 32% in Men	-	-	-	36, 46, 57, and 52% in Post-M, Peri-M, Pre-M, and Men, resp.	-	-	42, 50, 47, and 50% in Post-M, Peri-M, Pre-M, and Men, resp.	-	-	50, 30, and 44% in Post-M, Peri-M, and Pre-M, resp.	29% in Post-M and 25% in Pre-M/Peri-M	47% in both sexes	-

ACC, Anterior cingulate cortex; ApoE4, Apolipoprotein E 4; BA, Basal ganglia; BO, Bilateral oophorectomy; BG, Blood glucose; BMI, Body mass index; BP, Blood pressure; C, College; CHOL, Cholesterol; CMR, Cardiometabolic risk factors; CS, Cross-sectional; DI, Drinking index; DLPFC, Dorsolateral prefrontal cortex; EC, Entorhinal cortex; FG, Fasting glucose; FL, Frontal lobe; GM, Gray matter; HDL, High-density lipoprotein; HIP, Hippocampus; HRT, Hormone replacement therapy; HS, High school; HTN, Hypertension; HYS, Hysterectomy; inf., Inferior; IPL, Inferior parietal lobule; IR, Insulin resistance; lat., lateral; LDL, Low-density lipoprotein; LG, Longitudinal; M, Menopause; med., medial; MT, Menopause transition; O, Oophorectomy; PCC, Posterior cingulate cortex/precuneus; Peri-M, Perimenopausal women; pHYS, Partial hysterectomy; Post-M, Postmenopausal women; Pre-M, Premenopausal women; PFC, Prefrontal cortex; PHC, Parahippocampus; PL, Parietal lobe; resp., respectively; RBV, Regional brain volume; SCV, Subcortical volume; SI, Smoking index; Sig., Significant; SM, Surgical menopause; STRAW + 10, Stages of Reproductive Aging Workshop + 10; sup., superior; TBV, Total brain volume; TG, Triglyceride; TL, Temporal lobe; UNI, University; W, Women; WTH, Waist to hip ratio; 1° ed., Primary education; 2° ed., Secondary education; and 3° ed., Tertiary education.

**Table 3:** The stages of reproductive aging workshop +10 staging system for reproductive aging in women.

Menarche						FMP (0)				
	**↓**					**↓**				
Stage	-5	-4	-3b	-3a	-2	-1	+1a	+1b	+1c	+2
Terminology	Reproductive				Menopausal transition		Postmenopause			
	Early	Peak	Late		Early	Late	Early		Late	
					Perimenopause					
Duration	Variable				Variable	1–3 years	2 years (1 + 1)		3–6 years	Remaining lifespan
Principal criteria										
Menstrual cycle	Variable to regular	Regular	Regular	Subtle changes in flow/length	Variable lengthPersistent ≥7-day difference in length of consecutive cycles	Interval of amenorrhea of ≥60 days				
Supportive criteria										
Endocrine										
FSH			Low	Variable^*^	↑ Variable^*^	↑ ≥ 25 IU/L^**^	↑ Variable^*^	Stabilizes		
AMH			Low	Low	Low	Low	Low	Very low		
Inhibin B				Low	Low	Low	Low	Very low		
Antral follicle count			Low	Low	Low	Low	Very low	Very low		
Descriptive characteristics										
Symptoms						Vasomotor symptomsLikely	Vasomotor symptomsMost likely			Increasing symptoms of urogenital atrophy

Source: Harlow et al. (2012). ^*^Blood draw on cycle days 2–5. ^**^Approximate expected level based on assays using current international pituitary standard. AMH. Anti-Müllerian hormone; FSH, Follicle-stimulating hormone; FMP, Final menstruation phase; ↑, Elevated.

#### 3.3.2. Menopause characteristics

We noted that additional characterization of postmenopausal participants, including age at menopause, duration after menopause, and type of menopause, was scarce. However, two studies reported the mean age at menopause (Goto et al., 2011; Schelbaum et al., 2021), while one study recruited women who had been in postmenopause for 5.5 ± 2.5 years (Kim et al., 2018). In addition, Rahman et al. (2020), Schelbaum et al. (2021), and Than et al. (2021) reported the percentage of participants who underwent oophorectomy and hysterectomy, but they did not elaborate on whether the surgery was performed before or after the natural age of menopause.

#### 3.3.3. Age effect

All postmenopausal women in this review ranged from 50 to 70 years of age. To minimize the age effects, the studies included age as a confounder during analysis (Mosconi et al., 2017, 2018; Kim et al., 2018; Rahman et al., 2020; Schelbaum et al., 2021), used it as a grouping factor at the sample level (Cowell et al., 2007; Goto et al., 2011), used age-matched counterparts (den Heijer et al., 2003; Seitz et al., 2019; Zhang et al., 2021), or measured the total brain volume (TBV) in age-restricted (< 58 years old) subgroup populations (Than et al., 2021). On the other hand, Lu et al. (2018) confirmed the effect of menopause by performing a correlation analysis between age and the brain regions of interest (ROIs) of samples from all women. The authors considered brain regions that were not correlated with age but that showed volume differences between groups to be an exclusive effect of menopause. Rather than controlling for the age variable, Than et al. (2021) examined the age-dependent effects of menopause on regional brain volumes. On the contrary, three studies did not consider age as a covariate (Sullivan et al., 2005; Cowell et al., 2007; Baek et al., 2019).

#### 3.3.4. Use of HRT

A history or current use of HRT among menopausal women varied among of the studies, and none of the studies specifically reported the types of HRT used. Some studies excluded women if they were current HRT users during the experiment (den Heijer et al., 2003; Mosconi et al., 2018; Seitz et al., 2019) or had a history of HRT use 1 month prior to the scans (Kim et al., 2018; Lu et al., 2018; Baek et al., 2019). Conversely, several studies included HRT users; the percentage of postmenopausal women actively taking HRT or past users was 41% (Sullivan et al., 2005), 46% (Cowell et al., 2007), 14% (Mosconi et al., 2017), 26% (Rahman et al., 2020), and 7% (Than et al., 2021); 11% for perimenopausal women (Rahman et al., 2020); and 1.7% for pre-/perimenopausal women (Than et al., 2021). One particular study that also included HRT users distinguished between current or past users, 4 and 27%, respectively (Schelbaum et al., 2021).

#### 3.3.5. Education levels

The participants included in the studies had ≥12 years of education (Sullivan et al., 2005; Cowell et al., 2007; Mosconi et al., 2017, 2018; Lu et al., 2018; Rahman et al., 2020; Schelbaum et al., 2021; Zhang et al., 2021). Baek et al. (2019) reported that 26 and 68% of postmenopausal women had completed primary and secondary education, respectively, and only 5% had completed a university-level education. They reported that 79% of premenopausal women had completed a university-level education. In one study, the percentage of participants with primary education was similar in both men and women (den Heijer et al., 2003). Seitz et al. (2019) matched the academic status from secondary until tertiary education for the pre-, peri-, and postmenopausal women as well as the men. Three studies did not provide the educational background of the participants (Goto et al., 2011; Kim et al., 2018; Than et al., 2021).

#### 3.3.6. Cardiometabolic risk factors

The cardiometabolic risk factors (CMR) include hypertension, hyperlipidemia, obesity, insulin resistance, smoking, and alcohol consumption. Several studies examined the CMR among the participants (den Heijer et al., 2003; Goto et al., 2011; Mosconi et al., 2017, 2018; Rahman et al., 2020; Schelbaum et al., 2021; Than et al., 2021). Six studies did not provide details on the CMR (Sullivan et al., 2005; Cowell et al., 2007; Kim et al., 2018; Lu et al., 2018; Baek et al., 2019; Zhang et al., 2021). In one study, the postmenopausal group had significantly more CMR than the pre-/perimenopausal group (Than et al., 2021); however, the much larger (7-fold) size of the former group could have contributed to this difference. In contrast, in one study, the cholesterol/high-density lipoprotein (HDL) ratio was lower in postmenopausal women compared with the pre- and perimenopausal women (Rahman et al., 2020). However, other studies reported similar CMR among the participants (Goto et al., 2011; Mosconi et al., 2017, 2018; Rahman et al., 2020; Table 2). Interestingly, in two studies, male subjects were hyperlipidemic and had higher insulin resistance compared with women from different reproductive phases (Mosconi et al., 2017, 2018).

#### 3.3.7. ApoE4 carrier status

The ApoE4 carrier status was determined in multiple studies. Interestingly, these studies reported no significant difference in the percentage of carriers between the groups (den Heijer et al., 2003; Mosconi et al., 2017, 2018; Rahman et al., 2020; Schelbaum et al., 2021; Than et al., 2021). The authors considered the ApoE4 status to be a covariate and adjusted for it as a confounder during analysis.

#### 3.3.8. Brain ROIs

Several studies investigated specific brain regions based on *a priori* hypotheses, including the frontal, parietal, temporal, prefrontal, anterior, and posterior cingulate cortices; the precuneus; the amygdala; the putamen; the hippocampus; and the parahippocampus (den Heijer et al., 2003; Sullivan et al., 2005; Cowell et al., 2007; Goto et al., 2011; Mosconi et al., 2017, 2018; Seitz et al., 2019; Schelbaum et al., 2021). These studies aimed to examine specific brain regions implicated in cognitive functions during menopause. The remaining studies explored GM alterations between groups with whole-brain analyses without predefining specific brain regions.

### 3.4. Structural neuroimaging

#### 3.4.1. TBV and GM and WM volumes

The TBV refers to the overall volume or size of the entire brain, which includes both the GM and WM. Two studies reported significant reductions in the TBV as well as global GM and WM volumes in postmenopausal women compared with premenopausal women after controlling for age (Kim et al., 2018; Than et al., 2021; Table 4). These findings were supported by subgroup analysis restricted to populations <58 years old, which showed an interaction between age and menopause, indicating that for every 1-year increase in age, postmenopausal women had three times lower average predicted volume than pre-/perimenopausal group. This association remained significant after adjusting for other factors such as the ApoE4 status, HRT use, and CMR for both GM volume and TBV but not for WM volume (Than et al., 2021).

**Table 4:** Findings from the volumetric neuroimaging studies.

Study	Population characteristics		Brain regions measured	Menopause effects on brain volumes	Covariates
	Group (*n*)	Mean ± SD age or (age range), years			
den Heijer et al. (2003)	Post-M (210)	70 ± 8	Hippocampal volume	Post-M < Men	-
	Men (202)	69 ± 8			
Sullivan et al. (2005)	Post-M (27)	(20–85)	Hippocampal volume	Post-M = Pre-M	ICV
	Pre-M (17)		GM and WM volumes in the temporal lobe		
Cowell et al. (2007)	Peri-M (5) and Post-M (15)	(50–72)	Lateral prefrontal volume	Post-M and Peri-M < Pre-M	ICV
	Pre-M (16)	(20–49)	Medial prefrontal volume	Post-M and Peri-M = Pre-M	
Goto et al. (2011)	Post-M in their 50s (59)	55.4 ± 2.7	Bilateral hippocampal volume	Post-M in their 50s < Pre-M	ICV
	Post-M in their 60s (49)	64.2 ± 2.6			
	Post-M in their 70s (17)	74.1 ± 3.0			
	Pre-M (46)	45.1 ± 2.9			
Mosconi et al. (2017)	Post-M (14)	57 (52–60)	GM and WM volumes in the posterior cingulate/precuneus, frontal, temporal, and parietal regions	Post-M and Peri-M < Men	ICV, age, education, and ApoE4
	Peri-M (13)	50 (40–56)			
	Pre-M (15)	48 (40–55)	GM and WM volume in the frontal regions	Post-M < Peri-M < Pre-M	
	Men (18)	52 (42–60)			
Lu et al. (2018)	Peri-M (25)	51.6 ± 1.63	GM volume in the left putamen, right pallidum, right inferior parietal gyrus, right superior frontal gyrus (orbital part), and right postcentral gyrus	Peri-M < Pre-M	Age and ICV
	Pre-M (32)	47.75 ± 1.55			
Kim et al. (2018)	Post-M (20)	55.7 ± 2.4	Total GM and WM volumes	Post-M < Pre-M	Age
	Pre-M (20)	39.9 ± 8.1	GM volume in the inferior frontal gyrus, supplementary motor area, superior temporal gyrus, and olfactory cortex		
Mosconi et al. (2018)	Post-M (12)	58 ± 2 (55–60)	Rate of hippocampal volume change	Post-M: ↑ volume loss	ICV, age, education, ApoE4 status, and CMR
	Peri-M (14)	53 ± 4 (45–60)			
	Pre-M (15)	47 ± 5 (40–55)		Peri-M, Pre-M, and Men: minimal to no change	
	Men (18)	52 ± 6 (42–60)			
Baek et al. (2019)	Post-M (19)	55.5 ± 2.6	GM volume in the insula, putamen, parahippocampal gyrus, amygdala, and anterior cingulate gyrus	Post-M < Pre-M	-
	Pre-M (19)	40.2 ± 6.7			
Seitz et al. (2019)	Post-M (32)	50.59 ± 2.23	Volume of the hippocampus, anterior cingulate cortex, inferior parietal cortex and dorsolateral prefrontal cortex	Post-M = Peri-M = Pre-M	Age
	Peri-M (29)	49.83 ± 1.91			
	Pre-M (33)	49.24 ± 1.71			
Rahman et al. (2020)	Post-M (41)	58 ± 3	GM volume of the hippocampus, parahippocampal gyrus, amygdala, insula, and caudate	Post-M < Peri-M < Pre-M < Men	Age and ICV
	Peri-M (28)	51 ± 4			
	Pre-M (16)	44 ± 4	Regional WM volumes		
	Men (36)	52 ± 8			
Than et al. (2021)	Post-M (1,827)	63.9 (54.2–73.6)	TBV and GM and WM volumes	Post-M < Pre-M/Peri-M	Covariates for age and menopause interaction: ApoE4, CMR, and HRT
			TBV and GM volume	Post-M < Pre-M/Peri-M	
			Volumes of the frontal, temporal, parietal, occipital, and opercular cortices, and the paracingulate gyrus		
	Post-M (1,827)	63.9 (54.2–73.6)	WM volume	Post-M = Pre-M/Peri-M	
			Putamen	Post-M > Pre-M/Peri-M	
Schelbaum et al. (2021)	Post-M (49)	W: 52 ± 6 (40–65)	GM volume in the fusiform gyrus, amygdala, hippocampus, parahippocampus, and frontal and temporal regions	Post-M and Peri-M < Men	Age, ICV, hysterectomy status, and HRT use
	Peri-M (35)				
	Pre-M (15)	M: 52 ± 7 (40–65)	GM volume in frontotemporal regions	Post-M and Peri-M < Pre-M	
	Men (29)				
Zhang et al. (2021)	Peri-M (45)	47.38 ± 1.65	Amygdalar volume	Peri-M < Pre-M	Age and education level
	Pre-M (54)	46.89 ± 1.69	Hippocampal and basal ganglia volumes	Peri-M = Pre-M	

ApoE4, Apolipoprotein E4; CMR, Cardiometabolic risk factors; GM, Gray matter; HRT, Hormone replacement therapy; ICV, Intracranial volume; M, Men; Peri-M, Perimenopausal women; Post-M, Postmenopausal women; Pre-M, Premenopausal women; SD, Standard deviation; TBV, Total brain volume; W, Women; WM, White matter; >, more than; <, less than; =, equal to.

#### 3.4.2. Cortical structures

##### 3.4.2.1. Frontal regions

The frontal region undergoes structural changes during the menopausal transition, with reductions in GM and WM volumes (Table 4). Mosconi et al. (2017) found that both GM and WM volumes of the frontal region decreased significantly with each progression of the menopausal phase and relative to age-matched men. In the frontal region, there were volume differences in the orbital part of the right superior frontal gyrus, the lateral prefrontal cortex, the anterior cingulate cortex, the superior and inferior frontal gyri, and the supplementary motor area (SMA; Cowell et al., 2007; Kim et al., 2018; Lu et al., 2018; Baek et al., 2019; Schelbaum et al., 2021). The authors found these differences between peri- and postmenopausal women compared with premenopausal women and men. In another study, the authors found that the interaction between the menopause status and age further influences the association between age and the paracingulate gyrus volume in postmenopausal women (Than et al., 2021). However, some studies did not find significant differences in the anterior cingulate and dorsolateral prefrontal cortex volume (Seitz et al., 2019) as well as in the medial prefrontal volume among women, stratified by menopausal status (Cowell et al., 2007), indicating the complexity and variability of the effects of menopause on the frontal region. These findings collectively suggest that menopause is associated with marked volume differences in the frontal region.

##### 3.4.2.2. Temporal regions

Several studies investigated the impact of menopause on the temporal cortex. In one study, women had a smaller temporal cortex volume than men, and this effect was more evident in peri- and postmenopausal women (Mosconi et al., 2017). Specifically, the included studies reported that postmenopausal women have smaller volumes in the superior temporal gyrus, olfactory cortex, the entorhinal cortex, the fusiform gyrus, the superior and inferior temporal gyri, and the parahippocampus compared with premenopausal women (Kim et al., 2018; Baek et al., 2019;Rahman et al., 2020; Schelbaum et al., 2021). Furthermore, Than et al. (2021) reported an interaction between the menopause status and age: Postmenopausal women had a smaller temporal volume than pre-/perimenopausal women. However, Sullivan et al. (2005) did not find differences in the temporal lobe volume between pre- and postmenopausal women. These findings suggest that menopause can have significant effects on the temporal cortex.

##### 3.4.2.3. Parietal regions

In a large cohort study, an interaction between the menopause status and age influenced volume reductions in parietal regions, with postmenopausal women exhibiting lower volumes compared with the pre−/perimenopausal group (Than et al., 2021). Both peri- and postmenopausal women experience changes in different subregions of the parietal cortex. The posterior cingulate cortex and precuneus were smaller in peri- and postmenopausal women compared with age-matched men (Mosconi et al., 2017; Schelbaum et al., 2021). Additionally, the right postcentral and right inferior parietal gyri were reduced in perimenopausal women compared with premenopausal woman (Lu et al., 2018). On the contrary, Seitz et al. (2019) reported no volumetric difference in the inferior parietal lobule among women of different menopausal statuses. These findings suggest that menopause may contribute to structural changes in the parietal cortex, potentially impacting cognitive functions associated with this region.

##### 3.4.2.4. Occipital regions

There has been limited attention regarding menopause-related structural changes to the occipital cortex, which is responsible for visual processing and perception. Nevertheless, Than et al. (2021) observed a steeper negative association between age and occipital volume in postmenopausal women compared with pre-/perimenopausal women.

##### 3.4.2.5. Insula and opercular regions

The insula and opercular regions are adjacent brain structures with diverse functions including interoception, emotion processing, and sensorimotor integration (Gogolla, 2017). Although they have not been as extensively studied in the context of menopause compared with other brain regions, there is evidence suggesting structural changes in these areas. Both peri- and postmenopausal women exhibited lower volumes in the insula compared with premenopausal women and men (Baek et al., 2019; Rahman et al., 2020). Moreover, Than et al. (2021) described that the menopause status interacted with age, resulting in a lower opercular cortex volume in postmenopausal women compared with pre−/perimenopausal women.

#### 3.4.3. Subcortical structures

##### 3.4.3.1. Hippocampus

The hippocampus, a vital structure in the subcortical region of the brain, has been widely studied in the context of menopause. Several studies have investigated the impact of menopause on the hippocampal volume, and the authors have reported variable findings. In support of our hypothesis, both cross-sectional and longitudinal studies have reported significant differences in hippocampal volume between groups. Initial work by den Heijer et al. (2003) reported that older women had a significantly lower mean hippocampal volume than men. Further supporting these findings, Goto et al. (2011) demonstrated a smaller bilateral hippocampal volume in postmenopausal women compared with premenopausal women. Interestingly, Goto et al. (2011) also found that after menopause, the hippocampal volume remained relatively stable, showing minimal changes from the 50 to 70 s. Moreover, postmenopausal women had the smallest hippocampal volume, followed by peri- and premenopausal women, while men had the largest volume (Rahman et al., 2020; Schelbaum et al., 2021). In the longitudinal study, postmenopausal women had the highest rate of hippocampal volume atrophy. Over the 3-year of follow-up, there was an average of 3.3% hippocampal atrophy in postmenopausal women, while there was <1% atrophy in the other groups (Mosconi et al., 2018). However, several studies did not observe any significant difference in the hippocampal volume depending on the menopause status (Sullivan et al., 2005; Seitz et al., 2019; Zhang et al., 2021).

##### 3.4.3.2. Basal ganglia

The basal ganglia is a group of subcortical structures that comprises the caudate, putamen, and pallidum. Supporting our hypothesis, three studies reported smaller putamen volumes in peri- and postmenopausal women compared with premenopausal women and men (Lu et al., 2018; Baek et al., 2019; Schelbaum et al., 2021). Additionally, when comparing women of different reproductive phases, the caudate volume was lowest in postmenopausal women and lower relative to men (Rahman et al., 2020). Nevertheless, Zhang et al. (2021) did not observe any significant difference in the caudate, putamen, and pallidum volumes between peri- and premenopausal women. Interestingly, Than et al. (2021) reported a higher putamen volume in postmenopausal women than in pre-/perimenopausal women. These findings suggest complex and varied alterations in the volumes of specific basal ganglia structures during menopause.

##### 3.4.3.3. Amygdala

Several studies have examined the impact of menopause on the amygdalar volume. Supporting our hypothesis, multiple independent studies have demonstrated a significantly smaller amygdalar volumes in peri- and postmenopausal women compared with premenopausal women and men (Baek et al., 2019; Rahman et al., 2020; Schelbaum et al., 2021; Zhang et al., 2021).

### 3.5. Neuropsychological measures

Several of the included studies explored cognitive performance through various neuropsychological tools, yielding intriguing findings. The researchers employed verbal memory tests including paired associates delayed recall, paragraph recall, and word recall. In addition, they conducted visuospatial tasks through block design tests. However, verbal memory tests are particularly relevant and consistently revealed worse performance in postmenopausal compared with pre- and perimenopausal women (Table 5). After adjusting for age and education, Rahman et al. (2020) found that postmenopausal women scored lower in paired associates delayed recall tests than pre- and perimenopausal women. Further supporting this finding, postmenopausal women had higher cognitive decline rates than the pre- and perimenopausal women and men in the paragraph recall and block design tests in the longitudinal study (Mosconi et al., 2018). Conversely, den Heijer et al. (2003) observed a higher number of words recalled in women compared with men.

**Table 5:** Assessments of neuropsychological measures and brain volumetric changes.

Study	Neuropsychological tests	Menopause effects on cognition	Relationship between cognition and brain volume in Peri-M/Post-M
den Heijer et al. (2003)	Delayed recall	Post-M > Men	↑ Delayed recall scores but ↓ hippocampal volume
Mosconi et al. (2017)	DSSS	Peri-M and Post-M > Men	Cognitive function unaffected but ↓ GM volume in the frontal, posterior cingulate, precuneus, temporal, and parietal regions
	Paired associates delayed recall	Post-M = Peri-M = Pre-M = Men	
	Paragraph delayed recall		
	Designs score		
	Object naming		
Mosconi et al. (2018)	Paragraph recall	Post-M and Peri-M < Men	↓ Paragraph recall scores and block design tasks with ↓ hippocampus volume
	Block design	Post-M < Pre-M, Peri-M, and Men	
Rahman et al. (2020)	Paired associates delayed recall	Post-M < Pre-M and Peri-M	↓ Paired associates delayed recall scores with ↓ GM and WM volumes
	DSSS	Post-M = Peri-M = Pre-M = Men	
	Paragraph immediate and delayed recall		
	Designs score		
	Object naming		
	Vocabulary score		
Schelbaum et al. (2021)	RAVLT	Post-M = Peri-M = Pre-M = Men	Cognitive function unaffected but ↓ GM volume in the medial temporal lobe
	WMS-LM delayed recall test		
	Trail Making Test Part B		
	Object naming		
Zhang et al. (2021)	Two-back task	Peri-M < Pre-M	↓ Reaction time and accuracy rate in both tests with ↓ amygdalar volume
	Stroop test		

ACC, Anterior cingulate cortex; DSSS, Digit symbol substitution score; GM, Gray matter; Peri-M, Perimenopausal women; Post-M, Postmenopausal women; Pre-M, Premenopausal women; RAVLT, Rey auditory verbal learning test; WAIS, Wechsler adult intelligence scale; WM, White matter; WMS-LM, Wechsler Memory Scale logical memory; >, more than; <, less than; =, equal to; ↑, increased; ↓, decreased.

Apart from verbal memory tests, Zhang et al. (2021) explored executive function and working memory performance among perimenopausal women using the Stroop test and the two-back task, respectively. The study revealed that perimenopausal women showed a lower accuracy and a longer reaction time than premenopausal women in both of these tests.

On the other hand, Mosconi et al. (2017) reported that women at different reproductive stages and age-matched men had comparable performance in various neuropsychological tools, including paired associates delayed recall, paragraph delayed recall, designs score, object naming, and the Wechsler Adult Intelligence Scale (WAIS) vocabulary. The only significant findings were the digit symbol substitution scores: Men scored lower than the other groups. Similarly, Rahman et al. (2020) observed no effects of menopause in the tests mentioned above except for paired associates delayed recall. Moreover, Schelbaum et al. (2021) measured memory and global cognitive scores assessing executive function and language, which were not affected by the menopause status. However, the authors found significant correlations between the medial temporal region volume and the memory and cognitive scores.

### 3.6. Overall scoring of the evidence

Table 6 summarizes the evidence from the 14 included publications regarding the effects of the menopause status on each structural and neuropsychological parameter. Studies that reported negative impacts of both peri- and postmenopause on each parameter are scored −1, while positive or no effects are scored +1 and 0, respectively. The sum of all the scores within each parameter is shown in the overall score column. The denominator in the overall score represents the total number of studies that measured the effects of menopause on the respective parameters. We inferred a negative effect of menopause when the proportion of the summed score is at least more than half of the denominator. As depicted in Table 6, the menopause status impacted the TBV; the global GM and WM volumes; the frontal, temporal, parietal, and insular cortical volumes; and the hippocampal, basal ganglia, and amygdalar volumes. In addition, menopause had an inconsistent influence on memory tests, while its effect on visuospatial abilities and executive functions was limited.

**Table 6:** Brain structure and neuropsychological measures outcome in menopause.

	den Heijer et al. (2003)	Sullivan et al. (2005)	Cowell et al. (2007)	Goto et al. (2011)	Mosconi et al. (2017)	Mosconi et al. (2018)	Kim et al. (2018)	Lu et al. (2018)	Baek et al. (2019)	Seitz et al. (2019)	Rahman et al. (2020)	Than et al. (2021)	Schelbaum et al. (2021)	Zhang et al. (2021)	Overall score
A. Structural parameters															
1. TBV and GM volumes	.	.	.	.	.	.	−1	.	.	.	.	−1	.	.	−2/2^*^
2. WM volumes	.	.	.	.	.	.	−1	.	.	.	.	0	.	.	−1/2^*^
3. Cortical volumes															
*(i) Frontal regions*	.	.	−1	.	−1	.	−1	−1	−1	0	.	−1	−1	.	−7/8^*^
*(ii) Temporal regions*	.	0	.	.	−1	.	−1	.	−1	.	−1	−1	−1	.	−6/7^*^
*(iii) Parietal regions*	.	.	.	.	−1	.	.	−1	.	0	.	−1	−1	.	−4/5^*^
*(iv) Occipital regions*	.	.	.	.	.	.	.	.	.	.	.	−1	.	.	−1/1^*^
*(v) Insula and opercular regions*	.	.	.	.	.	.	.	.	−1	.	−1	−1	.	.	−3/3^*^
4. Subcortical volume															
*(i) Hippocampus*	−1	0	.	−1	.	−1	.	.	.	0	−1	.	−1	0	−5/8^*^
*(ii) Basal ganglia (pallidum/putamen/caudate)*	.	.	.	.	.	.	.	−1	−1	.	−1	+1	−1	0	−3/6^*^
*(iii) Amygdala*	.	.	.	.	.	.	.	.	−1	.	−1	.	−1	−1	−4/4^*^
B. Neuropsychological measures															
1. Memory (immediate and delayed recall of a paragraph, paired associates, and two-back task)	+1	.	.	.	0	−1	.	.	.	.	−1	.	0	−1	−2/6
2. Visuospatial (block design test)	.	.	.	.	.	−1	.	.	.	.	.	.	.	.	−1/1^*^
3. Executive function (Stroop test)	.	.	.	.	.	.	.	.	.	.	.	.	.	−1	−1/1^*^
4. Other tests	.	.	.	.	0	.	.	.	.	.	0	.	0	.	0/3

* Menopause is concluded to have a negative impact on the structural and neurophysiological parameters if the proportion of the summed score is at least more than half of the denominator for each parameter.

−1: negative effects of menopause on structural or neuropsychological measures; 0: no effects of menopause on structural or neuropsychological measures; +1: positive effects of menopause on structural or neuropsychological measures. GM, Gray matter; TBV, Total brain volume; and WM, white matter.

## 4. Discussion

This review of the 14 eligible neuroimaging studies revealed volumetric brain differences in menopausal women. Although the effects of aging on the brain have been explored extensively, there have been few studies investigating the impact of ovarian aging on volumetric changes in the brain. It can be challenging to distinguish between the effects of chronological versus reproductive aging because the two processes are tightly interrelated. However, alteration in the hormonal environment during menopause may significantly influence the structure of specific brain regions. We found that volumetric brain alterations during the perimenopausal phase were not temporary: They progressed further in the postmenopausal phase (Mosconi et al., 2018). The most frequently observed volume differences are in the frontal cortex followed by the hippocampus and the temporal cortex. These regions have long been known to play a central role in various behavioral and cognitive functions (Rubin et al., 2014; Sigurdsson and Duvarci, 2016; Armstrong et al., 2020). Consistently, the authors used memory-related tasks to assess the cognitive function of menopausal women, although they reported mixed findings. While there is impairment in visuospatial ability and executive function, these tasks were only measured in one study and, therefore, provided a weak relationship.

### 4.1. Brain volume

We found consistent evidence for brain volume alterations, especially in the frontal, hippocampal, and temporal regions of postmenopausal women. Importantly, these regions—consisting of the prefrontal cortex, hippocampus, and the temporal area—form memory circuit regions (Warburton and Brown, 2010; Huijgen and Samson, 2015; Chen et al., 2016). Seven out of eight studies uniformly reported volume alterations in the frontal regions in postmenopausal women, indicating the most affected areas in menopausal women. There are inconsistencies between reports on the hippocampal volume whereby some authors reported that the menopause status did not cause volume differences. However, the majority of the articles (five out eight) supported the negative effects of menopause on hippocampal volume. In addition, multiple studies found differences in the global GM volumes and other brain regions, including the parietal cortex, the insula, the basal ganglia, and the amygdala.

The reduced volume in the frontal regions is consistent with changes reported in preclinical menopause models: decreased spine density in the dorsolateral prefrontal cortex (Hao et al., 2007) and on the pyramidal neurons of the medial prefrontal cortex (Wallace et al., 2006). Some researchers have asserted that the nature of cognitive decline in postmenopausal women is primarily a deficit in executive function (Keenan et al., 2001; Joffe et al., 2006). In functional neuroimaging studies, researchers have hypothesized that postmenopausal women require more effort to maintain normal cognitive performance (denoted by higher activity in the frontal regions) or exhibit changes in neural connectivity (Jacobs et al., 2016; Vega et al., 2016). However, it is not entirely clear whether the structural alterations precede functional impairment in menopause. Nevertheless, increased spontaneous neuronal activity in the frontal regions and reduced GM volume in the left gyrus rectus have been observed in perimenopausal women (Liu et al., 2021).

The hippocampus is vital for learning, memorizing, and encoding new information into long-term memory; hence, damage or atrophy in this area has clinical consequences (Urgolites et al., 2020). A decreased GM volume in the right medial temporal lobe among menopausal women is associated with subjective cognitive complaints (Conley et al., 2020). Furthermore, studies in ovariectomized animals have shown significant alterations in the structure and function of hippocampus, followed by poor memory performance (Su et al., 2012; Sbisa et al., 2017; Xiao et al., 2018). Throughout the lifespan, the hippocampus exhibits structural plasticity that is implicated in aging, disease, and physiological regulation (Bartsch and Wulff, 2015). A form of structural plasticity that occurs in the hippocampus is adult neurogenesis (Toda et al., 2019). Neurons in the subventricular zone of the dentate gyrus retain the capacity to divide (Gonçalves et al., 2016). Researchers have reported impaired hippocampal neurogenesis in ovariectomized rats; it could be reversed with estradiol treatment (Tanapat et al., 1999; Ormerod et al., 2003; Barha et al., 2009). However, while day 6 post-ovariectomy resulted in diminished neurogenesis (Tanapat et al., 1999), this effect was short lived as evaluation at day 28 post-ovariectomy revealed no effect on neurogenesis (Tanapat et al., 2005). These results indicate that there may be a compensatory mechanism that restores neurogenesis at longer periods post-ovariectomy. This is further corroborated by a transcriptomic findings where long-term ovariectomy upregulated the genes involved in neurogenesis (Sárvári et al., 2016). It is possible that the proposed explanation for hippocampal volume preservation in postmenopausal woman might be related to neurogenesis that occurs years later after the FMP.

The smaller volume in the temporal regions are consistent with functional imaging studies that have reported reduced cerebral blood flow to the temporal regions (Słopień et al., 2003), reduced resting neuronal activity of the superior temporal gyrus (Liu et al., 2021), regional homogeneity in the inferior temporal gyrus (Zhang et al., 2022), and reduced glucose metabolism in the middle and inferior temporal gyri of postmenopausal women (Mosconi et al., 2021). A decrease in the inferior temporal gyrus volume affects visual perception, language comprehension, and verbal fluency (Lin et al., 2020), while a reduced superior temporal gyrus volume affects speech sounds, language function, and social cognition (Ramos Nuñez et al., 2020). In addition, a decrease in smell acuity among postmenopausal women might be explained by a volume loss in the olfactory cortex (Doty et al., 2015).

The pallidum, putamen, and caudate nucleus are the subcortical nuclei that form the basal ganglia, which is primarily involved in motor controls, although its role in cognitive functions has been well established (Hélie et al., 2015; Moretti et al., 2017). Both the putamen and pallidum showed smaller volumes in menopausal women, although postmenopausal woman had a larger putamen volume than pre−/perimenopausal women (Table 6). Previous studies have reported structural changes in the basal ganglia in response to sex hormone fluctuations across the menstrual cycle. During the midluteal phase of the menstrual cycle, there is a positive correlation between the basal ganglia volume and progesterone levels (Pletzer et al., 2018). In addition, women who use oral contraceptives have a larger basal ganglia volume than women who do not (Pletzer et al., 2019). On the other hand, the basal ganglia volume is smaller when estradiol levels are relatively higher during the late follicular phase (Pletzer et al., 2018).

We noted negative impacts of menopause in all of the studies that measured the insular and amygdalar volumes (Table 6). The insula is important for cognition, decision-making, and somatosensory function (Uddin et al., 2017). Alterations in its structure are associated with various deficits, including speech and language processing as well as understanding emotions and behavior (Menon and Uddin, 2010). Despite its relatively small size, the amygdala contains abundant ERs that mediate the action of estradiol on emotions and memory (Framorando et al., 2021). Consistently, Engman et al. (2018) observed increased functional amygdalar connectivity in women with higher estradiol levels.

### 4.2. Neuropsychological tests

Six studies investigated whether the observed structural brain differences are supported by evidence of altered cognitive function. Neuropsychological tests are often used to assess behavioral changes and have been used to diagnose cognitive impairment in people with neurological diseases. The tests include a wide range of approaches that evaluate different parts of the cognitive domains. One of the tests of verbal memory assesses the recall of either verbal lists (den Heijer et al., 2003) or short paragraphs (Mosconi et al., 2017, 2018; Rahman et al., 2020). In the included studies, reduced verbal memory performance in postmenopausal women was associated with smaller brain volumes, notably in the hippocampus (Mosconi et al., 2018; Rahman et al., 2020). This observation corresponds with a growing body of evidence associating verbal memory and the hippocampus (Beyer et al., 2013; Seitz et al., 2019; Witt et al., 2019). Interestingly, den Heijer et al. (2003) observed that postmenopausal women recalled more words despite having a lower hippocampal volume than men. However, when the authors divided the postmenopausal women into three groups according to their total, bioavailable, and free estradiol levels, the group with the highest bioavailable and free estradiol levels had the lowest scores in the delayed recall test and the smallest hippocampal volume. Although the findings of this study do not support the neuroprotective effects of estradiol, the reduced hippocampal volume was associated with poor memory performance (den Heijer et al., 2003).

On the other hand, two studies did not observe differences in the verbal memory scores, although there were structural brain differences between groups (Mosconi et al., 2017; Schelbaum et al., 2021). The preservation of verbal memory despite structural brain alterations of postmenopausal women in these studies can be illustrated in the context of the cognitive reserve (CR) hypothesis. Women have greater advantages on verbal memory than men due to sex-specific CR in the domain of verbal memory (Beinhoff et al., 2008; Sundermann et al., 2016a,b) combined with estrogen-mediated modulation of hippocampal function, including synapse and spine formation, signaling, and excitability (Spencer et al., 2008; Shanmugan and Epperson, 2014). However, the drawback of having better verbal memory includes missing an amnestic mild cognitive impairment diagnosis and delayed AD detection even when brain pathological changes are present (Sundermann et al., 2019). This further explains why women present a more rapid decline across a wide range of cognitive abilities after being diagnosed with AD. Based on the current review, the extent of the verbal memory advantage women have is still unclear given the inconclusive findings among the studies. Future studies should explore the interaction between verbal memory and menopause and its correlation with regional brain volumes on a larger scale.

Other cognitive domains affected by menopause are working memory and executive function, as evidenced by reduced performance in the two-back task and the Stroop test, respectively (Zhang et al., 2021). Furthermore, these tests presented significant correlations with the amygdalar volume: The two-back task accuracy had a positive correlation while the Stroop test reaction time showed a negative correlation (Zhang et al., 2021). The coordination of several interconnected brain regions is crucial for working memory and executive function, with the prefrontal cortex playing a central role (Barbey et al., 2013). Prior studies have highlighted the functional relationship between the amygdala and the bilateral prefrontal cortex (Kim et al., 2010, 2011). Furthermore, alterations in functional connectivity between the amygdala and the bilateral prefrontal cortex have been observed in postmenopausal women; these changes were associated with decreased executive functions (Zhang S. et al., 2018).

Visuospatial ability refers to the cognitive process that enables a person to visually identify targets in space and to interpret the relationship of objects in the environment in more than one dimension (de Bruin et al., 2016). fMRI scans shows activation in the occipital-temporal and frontal–parietal networks during visuospatial tasks (Gogos et al., 2010). In the current review, postmenopausal women had the greatest rate of decline in the block design test of all the reproductive states and men (Mosconi et al., 2018). Moreover, postmenopausal woman had the greatest rate of decline in glucose metabolism in the frontal cortex, a finding suggesting that the decline in the block design test may be potentially associated with alterations in glucose metabolism in the frontal cortex (Mosconi et al., 2018). These findings further support previous reports indicating that the block design test, used to assess visuospatial skills, is sensitive to changes in estrogen levels, particularly in the context of menopause (Duka et al., 2000; Mosconi et al., 2018; Karishma et al., 2020).

In contrast to verbal and working memory, executive function, and visuospatial ability, women of different reproductive states and men had similar scores in the WAIS-R object naming and vocabulary scores (Mosconi et al., 2017; Rahman et al., 2020). These subtests measure perceptual reasoning and the verbal comprehension index (Morin and Midlarsky, 2017; Scott et al., 2021). In addition, Schelbaum et al. (2021) reported no difference in global cognitive scores from which they assessed visual attention, task switching, language, and memory, although there was a positive correlation between the medial temporal lobe GM volume and the cognitive scores. The lack of score difference suggests that the preceding brain structural changes following menopause may not correlate with the cognitive performance in the aforementioned cognitive domains. This could be influenced by more education and a younger age despite menopause. Based on this information, brain structural changes seem to be more sensitive to the hormonal milieu, whereas cognitive performance deficits either manifest much later or may not be detectible with a single endpoint measure.

### 4.3. Effects of confounding variables

We had to consider several demographic variables across the studies, especially those that could significantly influence the outcome measures. These variables include age, age at menopause (early vs. late menopause), the type of menopause (natural vs. surgical), HRT use, education level, the CMR, and the ApoE4 carrier status. A large body of evidence indicates that there is an age-associated reduction in global and regional brain volumes (Ritchie et al., 2015; Schippling et al., 2017), and these changes have a relationship with cognitive performance (Persson et al., 2016; Nave et al., 2019). In this regard, all but two of the included studies controlled for age to differentiate the effects of chronological age from the effects of menopause status on brain volume. This suggests that menopause significantly influences the decline in global and regional brain volume independent of the aging process. Furthermore, menopause could accelerate brain aging, as shown by a steeper decline in the age-associated reduction in brain volumes of postmenopausal women compared with pre−/perimenopausal women (Than et al., 2021). However, the volume changes in brain regions after menopause varies. Kim et al. (2018) reported a negative correlation between the SMA volume and years after menopause, while Goto et al. (2011) showed no significant difference in the hippocampal volume when they compared postmenopausal women in their 50, 60, and 70 s.

The median age of natural menopause is 51 years, preceded by 4–10 years of perimenopause (Gold et al., 2013). Nevertheless, women could have premature or early menopause if their FMP occurs before 40 and 45 years of age, respectively, due to various causes, including surgical and non-surgical (Shuster et al., 2010). Early menopause is associated with shorter lifetime exposure to endogenous sex hormones, which negatively impacts neurological health (Matyi et al., 2019; Hao et al., 2023) and the cardiovascular system (Zhu et al., 2019). In contrast, prolonged exposure to endogenous hormones exerts neuroprotective effects, as evidenced by a larger GM volume of the superior parietal lobule and left precuneus (Schelbaum et al., 2021). Unfortunately, the included studies did not provide the participants’ age at the initiation of menopause. This lack of information may be attributed to several factors, including limited data availability and a focus on hormones or specific outcome measures rather than age at menopause when the authors designed their analyses. However, considering the relevance of age at menopause in relation to cardiovascular risk factors and the onset of dementia, it is crucial to include age at menopause as an important covariate in future studies.

Only three of the included studies included active HRT users (Sullivan et al., 2005;Cowell et al., 2007; Mosconi et al., 2017), while most of the included studies excluded them or controlled for HRT use. Studies that included postmenopausal women actively taking HRT showed smaller volumes in the frontal regions than premenopausal women and relative to men (Cowell et al., 2007; Mosconi et al., 2017), while the hippocampus and temporal lobe showed no volume differences between pre- and postmenopausal woman (Sullivan et al., 2005). The Women’s Health Initiative Memory Study (WHIMS-MRI) reported that HRT was associated with GM reduction in the hippocampal and frontal regions (Resnick et al., 2009; Zhang et al., 2016). On the other hand, the dorsolateral prefrontal cortex volume was preserved in postmenopausal women who used a transdermal estradiol patch compared with the placebo group (Kantarci et al., 2016). The inconsistencies regarding the HRT effects on the brain structures gave rise to a speculative theory, termed the critical window hypothesis, which suggests that HRT is beneficial in the years immediately after menopause but may be deleterious when initiated ≥10 years after menopause (Whitmer et al., 2011; Maki, 2013; McCarrey and Resnick, 2015). In the current review, the absence of data on the HRT initiation time, formulation, and duration makes it difficult to conclude whether HRT is deleterious to the frontal lobe or effective in preserving the temporal lobe. In this context, it is unclear whether HRT provides neuroprotection; this issues requires further investigation.

Six of the included studies described the number of participants with the ApoE4 genotype, of which their distribution was similar between the groups and controlled as confounders (Table 2). Therefore, the brain volume alterations of postmenopausal women in these studies are independent of the ApoE4 status. The ε4 allele of the *APOE* gene is known to be the strongest genetic risk factor associated with sporadic AD (Serrano-Pozo et al., 2021). Previous studies have examined the ApoE4 by sex interaction, suggesting that female carriers are at higher risk of developing and accelerated AD progression, cognitive impairment, and lower brain volumes than male carriers (Sampedro et al., 2015; Neu et al., 2017; O’Bryant et al., 2022). Nevertheless, the effects of HRT on brain volume seemed to be more beneficial in ApoE4 carriers, who have been reported to have higher hippocampal, entorhinal and amygdala volumes (Yue et al., 2007; Saleh et al., 2023). Interestingly, the advantageous effect of starting HRT early or during the critical window on the hippocampal volume was limited to ApoE4 carriers (Saleh et al., 2023). This finding suggests that ApoE4 carriers are more sensitive to an HRT intervention, especially if it is initiated early.

The recruited subjects had at least completed primary school and had an average of 12 years of education. This level of education ensures a more homogenous socioeconomic status by minimizing low education variability that may influence the strength of the association between the menopause status and brain volume (Table 2). Education is neuroprotective as illustrated in the context of the CR theory. Essentially, individuals with higher CR can withstand advanced pathogenic mechanisms of neurodegenerative diseases or brain damage without showing any clinical manifestations (Meng and D’Arcy, 2012; Amieva et al., 2014). Education is widely used as an indirect indicator of CR, although other determinants such as occupational complexity, intelligence, and the participation rate in cognitively stimulating activities have also been considered. A cognitively stimulating environment promotes neurogenesis (Brown et al., 2003) and synaptic plasticity, and upregulates BDNF levels in animal models (Zhang M. et al., 2018). With evidence regarding the neuroprotective role of CR, future studies investigating its association with the structural and functional brain in menopausal women are needed to gain insight into predicting dementia risk.

Eight of the included studies described various CMR, including clinical (e.g., blood pressure, lipid profiles, obesity, glucose levels, and insulin resistance) and non-clinical (e.g., smoking and alcohol consumption) indicators (Table 2). Prior studies have reported clear evidence on the association between hypertension, hyperglycemia, and central obesity and brain structure alterations including smaller GM and total brain volumes, a thinner cortex, and larger ventricles than healthy controls (Song et al., 2020; Vergoossen et al., 2020). In the current review, while men had higher lipid levels and insulin resistance, they showed larger regional brain volumes than women after correcting for the intracranial volume (ICV; Mosconi et al., 2017, 2018; Rahman et al., 2020). Conversely, postmenopausal women who had lower lipid levels displayed smaller regional brain volumes than the pre- and perimenopausal women (Rahman et al., 2020). Concurrently, although there was no difference in the CMR among the pre-, peri-, and postmenopausal groups, the latter exhibited the lowest volume in the frontal and hippocampal regions (Goto et al., 2011; Mosconi et al., 2017, 2018). It is important to note that the participants included in the studies were healthy, independent of clinical diagnoses of cardiometabolic diseases. Additional studies with larger samples that include various degrees of cardiometabolic diseases and their risk factors are needed to investigate its influence on the brain structure of menopausal women.

### 4.4. Methodological considerations

Based on the included studies, the frontal and temporal cortices and the hippocampus are the most consistently reported regions with changes based on ROI-based (hypotheses-driven) and whole-brain analyses (Table 6). Studies based on *a priori* hypotheses may be biased toward findings in brain regions that have previously been associated with aging- and AD-related ROI, while possible associations with other regions of the brain may be overlooked. Whole-brain studies overcome this problem, but subtle differences may go unnoticed due to strict adjustment for multiple comparisons. Indeed, three hypothesis-driven studies did not find volume differences in the hippocampus (Sullivan et al., 2005; Seitz et al., 2019; Zhang et al., 2021), as observed in exploratory whole-brain studies. These inconsistent findings may indicate that MRI is not sensitive enough to detect subcortical volume alterations or that other factors might influence to preserve the hippocampal volume independent of the menopause status.

The frontal, temporal, and parietal regions were the most frequently measured cortical volumes, reported in eight, seven, and five studies, respectively (Table 6). Interestingly, the impact of menopause on the frontal, temporal, and parietal subregions was not uniform across studies (Table 4). Each subregion of the cortex has a distinct cytoarchitecture that represents its functionality (Brodmann, 1909; Amunts et al., 1999). As such, higher cognitive tasks, including executive function, working memory, inhibitory control, and cognitive flexibility, depend on the integrity of the frontal cortex (Garcia-Alvarez et al., 2019). On the other hand, the temporal cortex has vast functions, with at least eight cognitive domains identified related to speech, hearing, visual, episodic memory, phonological processing, semantic, and social cognition (Bajada et al., 2017), while the parietal cortex is involved primarily in receiving and integrating sensory inputs (Freund, 2003). For MRI, the common approach to defining the parcellation in the brain is to use a variety of atlases spatially normalized to the stereotactic space (Thyreau and Taki, 2020; Lawrence et al., 2021). However, different parcellations across studies make assessing the reproducibility of menopause-related brain changes difficult. In the current review, three studies used manual parcellation (den Heijer et al., 2003; Sullivan et al., 2005; Cowell et al., 2007), while the remaining studies utilized various atlas-based methods or parcellation protocols (Goto et al., 2011; Mosconi et al., 2017, 2018; Kim et al., 2018; Lu et al., 2018; Baek et al., 2019; Seitz et al., 2019; Rahman et al., 2020; Schelbaum et al., 2021; Zhang et al., 2021). Challenges associated with different anatomical parcellations include inter-subject variability, nomenclature problems, and inconsistency in identifiable landmarks (Bohland et al., 2009; Moghimi et al., 2021). Therefore, standardized parcellation protocols across different populations would provide researchers with a valuable resource to evaluate future neuroimaging studies and serve as a guide for better interpretation (Bohland et al., 2009; Mandal et al., 2012).

Due to the small sample sizes of the included studies, most authors employed various correction methods for multiple comparisons, including the Bonferroni, false discovery rate, familywise error, bootstrapping, and cluster-level small volume corrections. However, three of the studies did not indicate the specific correction methods used (den Heijer et al., 2003; Sullivan et al., 2005; Cowell et al., 2007). Additionally, one study examined the results at a *p* < 0.001 threshold for a specific brain region based on *a priori* hypotheses without implementing any correction for multiple comparisons (Mosconi et al., 2017). Overall, the included studies from 2011 to 2021 recognized the importance of multiple comparison correction to reduce the risk of false positives. This recognition enhanced the reliability and validity of study findings, thereby improving study generalizability.

## 5. Limitations

Our review has several limitations. First, we could not completely rule out the effects of HRT use among the participants because most of the studies included HRT users (past or active); this inclusion may confound the association between menopause and brain volumes. Second, the age range of the postmenopausal women varied among the studies, which could explain the different outcomes regarding brain volume differences and the regions involved. Some studies recruited young postmenopausal women, while others used elderly postmenopausal participants. This is important because elderly postmenopausal women could have more structural alterations that may be compounded by chronological age. Third, while the majority of the studies used the STRAW+10 criteria to classify the menopause stage, there was a lack of sex hormone measurement to support the menopause status in most studies. Fourth, to allow for a uniform analysis, brain regions for the structural parameters in the evidence scoring table were allocated according to the classic anatomical lobe classification (frontal, temporal, parietal, and occipital), even though the specific subregions within the lobes were not similar across different studies. Fifth, we could not determine the reliability of structural and neuropsychological parameters that were only assessed in one study. Sixth, due to the small number of studies that measured cognitive performance and the few cognitive domains tested, we could not draw meaningful conclusions regarding the relationship between the effects of menopause and neuropsychological measures. Seventh, the predominant cross-sectional study design of the eligible studies does not allow determining causal or temporal relationships.

Future studies should include a thorough description of the types, duration, and initiation of HRT use; measurement of circulating ovarian hormone levels; control for age effects; standardization of experimental methods; a longitudinal study design in different ethnic populations; and correlation of the MRI data to sex-specific cognitive function. In addition, instead of focusing on specific subregions, whole-brain analysis could uncover more regions that are susceptible to menopause-related changes.

## 6. Perspectives and conclusion

Across the included studies, there is compelling evidence for menopause effects on the cortical and subcortical brain regions that are key to cognitive processes. It is challenging to state that there is a strong association between the menopause status and brain volume differences because of the inconsistencies in the measured brain regions, the small number of eligible studies, and the cross-sectional nature of most of the studies. However, based on the evidence scoring table, ROI analyses have most frequently highlighted the involvement of the frontal and temporal regions. Additionally, the hippocampus has consistently emerged as a prominent affected area. In addition, memory-related tasks have been the most used task when assessing cognitive function in menopausal women, although there were inconsistent findings. The visuospatial ability and executive function tasks were only measured in one study and, therefore, provided a weak relationship. Although the current literature is limited by the heterogeneity in population characteristics, menopause-associated factors, and the potential confounding variables, it will be essential to conduct large, well-designed prospective neuroimaging studies to identify volumetric brain changes over time at the regional and whole-brain levels to draw stronger conclusions. Comprehensive and reliable neuroimaging findings, especially on specific subregions that are most prone to the effects of menopause, could provide a basis for additional related research especially on the neurobiological pathway mechanisms. Furthermore, providing coordinated data from a standardized stereotaxic space in neuroimaging studies could be used for a future meta-analysis.

## Author contributions

NR, HD, MY, and HA contributed to the study conception. NR designed the methodology. NR, HD, and MY conducted formal analysis and wrote the original draft. HA, MY, NM, and MS provided comments and revisions to the draft. HD supervised the research activity and managed the funding sources. All authors contributed to the article and approved the submitted version.
